# A mid-term assessment of progress towards the immunization coverage goal of the Global Immunization Vision and Strategy (GIVS)

**DOI:** 10.1186/1471-2458-11-806

**Published:** 2011-10-14

**Authors:** David W Brown, Anthony Burton, Marta Gacic-Dobo, Rouslan I Karimov, Jos Vandelaer, Jean Marie Okwo-Bele

**Affiliations:** 1 United Nations Children's Fund, New York, New York, USA; 2 World Health Organization, Geneva, Switzerland

**Keywords:** immunization coverage, statistics, immunization, monitoring, Global Immunization Vision and Strategy (GIVS)

## Abstract

**Background:**

The Global Immunization Vision and Strategy (GIVS) (2006-2015) aims to reach and sustain high levels of vaccine coverage, provide immunization services to age groups beyond infancy and to those currently not reached, and to ensure that immunization activities are linked with other health interventions and contribute to the overall development of the health sector.

**Objective:**

To examine mid-term progress (through 2010) of the immunization coverage goal of the GIVS for 194 countries or territories with special attention to data from 68 countries which account for more than 95% of all maternal and child deaths.

**Methods:**

We present national immunization coverage estimates for the third dose of diphtheria and tetanus toxoid with pertussis (DTP3) vaccine and the first dose of measles containing vaccine (MCV) during 2000, 2005 and 2010 and report the average annual relative percent change during 2000-2005 and 2005-2010. Data are taken from the WHO and UNICEF estimates of national immunization coverage, which refer to immunizations given during routine immunization services to children less than 12 months of age where immunization services are recorded.

**Results:**

Globally DTP3 coverage increased from 74% during 2000 to 85% during 2010, and MCV coverage increased from 72% during 2000 to 85% during 2010. A total of 149 countries attained or were on track to achieve the 90% coverage goal for DTP3 (147 countries for MCV coverage). DTP3 coverage ≥ 90% was sustained between 2005 and 2010 by 99 countries (98 countries for MCV). Among 68 priority countries, 28 countries were identified as having made either insufficient or no progress towards reaching the GIVS goal of 90% coverage by 2015 for DTP3 or MCV. DTP3 and MCV coverage remained < 70% during 2010 for 16 and 21 priority countries, respectively.

**Conclusion:**

Progress towards GIVS goals highlights improvements in routine immunization coverage, yet it is troubling to observe priority countries with little or no progress during the past five years. These results highlight that further efforts are needed to achieve and maintain the global immunization coverage goals.

## Background

In response to challenges in global immunization following tremendous success experienced during the 1980s, the United Nations Children's Fund (UNICEF), the World Health Organization (WHO) and partners developed the Global Immunization Vision and Strategy (GIVS) for the years 2006-2015 [1,2]. The GIVS aims to assist countries to protect more people against diseases by expanding the reach of immunization to every eligible person, including those in age groups beyond infancy, within a context in which immunization is high on national health agendas. The global strategy comprises four strategic areas with 24 component strategies [1,2] from which countries can choose for implementation according to their specific needs. The four areas are: to immunise more people against more diseases; to introduce a range of newly available vaccines and technologies; to integrate immunization other critical health interventions; and to manage vaccination programmes within the context of global interdependence.

Among the goals and targets to further prevent morbidity and mortality from vaccine preventable diseases set forth by GIVS was to increase national immunization coverage to at least 90% by 2010 and to sustain such levels of immunization coverage through 2015. Using WHO and UNICEF estimates of national immunization coverage for the third dose of diphtheria, tetanus toxoid and pertussis vaccine (DTP3) and the first dose of measles containing vaccine (MCV), we report the number of countries or territories that have met the 90% goal by 2010 and progress towards the GIVS coverage goal for those that did not meet the goal by 2010.

## Methods

Each year since 2000, WHO and UNICEF have jointly reviewed, prepared and published estimates of national immunization coverage for select vaccines. A detailed explanation of the estimation methods is provided elsewhere [3]. Briefly, reports by national authorities to WHO and UNICEF and survey data from the published and grey literature are reviewed. Based on these data, with due consideration to potential biases and the views of local experts (primarily national immunization system managers and WHO/UNICEF regional and national staff), WHO and UNICEF jointly estimate the most likely immunization coverage levels for each country or territory. It is important to emphasize that while the WHO and UNICEF estimates are informed by data from national authorities and may not differ from official government reported data, they constitute an independent technical assessment by WHO and UNICEF of the national routine immunization system performance.

Immunization coverage levels are presented as the percentage of a target population that has been vaccinated. For example, DTP3 coverage is calculated by dividing the number of children receiving the third dose of DTP vaccine by the number of children who survived to their first birthday. It is important to emphasize that the WHO and UNICEF estimates of national immunization coverage refer to immunizations given during routine immunization services to children less than 12 months of age where such services are recorded; not included are supplementary immunization activities such as polio, tetanus and measles campaigns. The WHO and UNICEF estimates are not the result of a formal modeling exercise and no statistical or mathematical models are used, with the exception that coverage for the first dose of DTP vaccine (DTP1) is based on the result of a simple ordinary least squares model of the relationship between DTP1 and DTP3 in those instances where DTP1 data are missing or where a country reports DTP1 coverage below DTP3. While there are frequently general trends in immunization coverage levels, no attempt is made to fit data points with smoothing techniques or time series methods though the estimation process does allow for interpolation within the time series and extrapolation at the end of the time series. To better ensure the consistency and replicability of the estimates and the transparency of methods, WHO and UNICEF began representing the data, information and decision heuristics in 2010 using a formal knowledge representation and reasoning system, described in detail elsewhere [4].

We report global and regional (Millennium Development Goal regions; available at http://www.un.org/millenniumgoals/index.shtml) DTP3 and MCV coverage. Global and regional averages are obtained by multiplying the country-specific immunization coverage and a population weight for each country where the weight is the country-specific proportion of the global (or regional) total population. Population estimates of the number of surviving infants for each country are obtained from the United Nations Population Division [5].

We report the number of countries or territories that reached the 90% goal by 2010 and examine progress towards the GIVS coverage goal by 2015 for those countries that did not attain the goal by 2010. In addition to reporting for 194 countries or territories, we highlight the coverage data and GIVS progress for 68 priority countries where more than 95% of all maternal and child deaths occur. These 68 priority countries comprise a group focused on by the *Countdown to 2015: Tracking Progress in Maternal, Newborn and Child Survival *project, a global effort supported by academics, governments, international agencies, health care professional associations, donors and nongovernmental organizations [6]. The *Countdown *uses country-specific data to stimulate and support country progress towards achieving the health-related MDGs.

The GIVS coverage goal is measured here against DTP3 and MCV national coverage data for 2010. Countries were considered to have reached the objective if they attained 90% coverage by 2010. Progress towards the GIVS coverage goal by 2015 is based on national DTP3 and MCV coverage data for 2000, 2005 and 2010 and the average annual relative percent change during 2005-2010. For the purposes here, countries and territories were classified as "on track", having made "insufficient progress" or "no progress" with regards to the 90% GIVS coverage goal in a hierarchical manner as follows. For those with coverage levels < 90% for 2010, countries were considered "on track" for the objective for 2015 if the country had an average annual rate of increase for the period 2005-2010 greater than or equal to that necessary to reach 90% by 2015 from the 2010 coverage level. We classified countries as having made "insufficient progress" if the average annual rate of increase was less than that needed to reach 90% by 2015 for the period 2005-2010 or having made "no progress" if coverage decreased or there was no change in coverage for the period 2005-2010. Countries whose coverage level was 80-89% during 2010 but who had either no change in coverage or decreasing coverage during 2005-2010 were classified as having made "insufficient progress". We classified countries with coverage levels of 90% or greater during 2000 and 2005 but whose coverage during 2010 was < 90% on a case-by-case basis based on a review of the immunization coverage time-series. The computed average annual percent change was based on a linear relationship between the log of coverage and year during 2005-2010. All national coverage estimate data are based on the 2010 revision (July 2011) of the WHO and UNICEF estimates and are available online at http://www.who.int/immunization_monitoring/data/en/index.html and http://www.childinfo.org/immunization.html.

## Results

**Figure 1:**
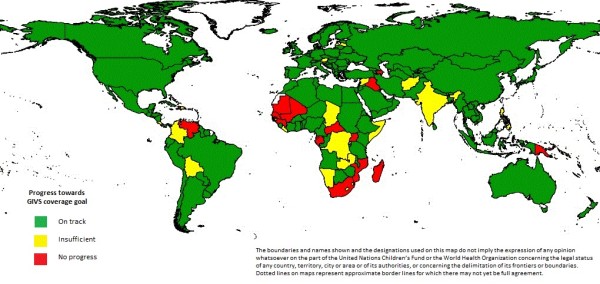
**Progress towards the Global Immunization Vision and Strategy (GIVS) 90% coverage goal for the third dose of diphtheria and tetanus toxoid with pertussis vaccine**.

**Figure 2:**
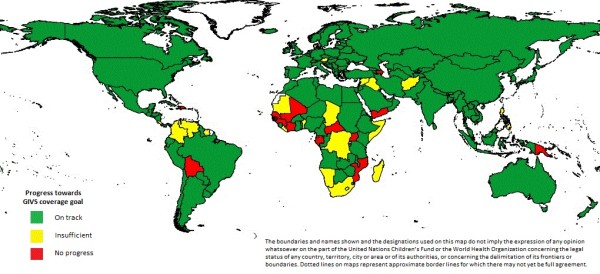
**Progress towards the Global Immunization Vision and Strategy (GIVS) 90% coverage goal for the first dose of measles containing vaccine**.

Globally DTP3 coverage increased from 74% during 2000 to 85% during 2010; global MCV coverage increased from 72% during 2000 to 85% during 2010 (Table 1). Progress towards the 90% GIVS coverage goal for all countries and territories for DTP3 is shown in Figure 1 and that for MCV is shown in Figure 2. More than three-quarters of countries achieved at least 90% coverage for DTP3 (DTP3, n = 149) or MCV (MCV, n = 148) during 2010, or were on track to achieve at least 90% coverage for DTP3 or MCV by 2015. Among 145 countries or territories who attained 90% DTP3 coverage at some point during the first five years of GIVS (2006-2010), DTP3 coverage ≥ 90% was sustained for five years by 99 countries; 118 countries achieved DTP3 coverage ≥ 90% for four of the five years and 125 countries achieved DTP3 coverage ≥ 90% for three of the five years. MCV coverage ≥ 90% was sustained during the five year period by 98 countries; 112 countries achieved MCV coverage ≥ 90% for four of the five years and 123 countries achieved MCV coverage ≥ 90% for three of the five years.

**Table 1:** Global and regional averages of WHO and UNICEF estimates of national routine immunization coverage (%) with three doses of diphtheria and tetanus toxoid with pertussis vaccine (DTP3) and with first dose of measles containing vaccine (MCV), 2000, 2005, 2010

	DTP3 coverage (%)			MCV coverage (%)		
MDG Region*	**2000**	**2005**	**2010**	**2000**	**2005**	**2010**

Sub-Saharan Africa	55	65	77	55	63	75
Northern Africa	95	96	97	93	95	96
Western Asia	84	84	86	85	83	85
Caucasus & Central Asia	93	93	94	93	94	94
Eastern Asia	85	87	99	84	87	99
South-Eastern Asia	80	82	88	81	84	91
Southern Asia	65	72	77	59	69	78
Oceania	65	66	62	66	66	59
Caribbean	73	78	79	76	77	76
Latin America	92	94	94	94	94	94
Developed	93	96	95	92	94	94
						
Global	74	79	85	72	78	85

* Millennium Development Goal Region, available at http://www.un.org/millenniumgoals/index.shtml

*Source*: WHO and UNICEF estimates of national routine immunization coverage, 2010 data revision (July 2011)

Nearly two-thirds of surviving infants unvaccinated with DTP3 during 2010 resided in one of 45 countries making either insufficient or no progress towards the GIVS coverage goal for DTP3 (these 45 countries accounted for one-third of surviving infants globally, 34.1% or 44,012,000 infants during 2010). Of these 45 countries, 41 countries were developing or least developed countries (according to World Bank classification [7]) and 22 countries were located in Africa; similar results were observed for MCV (42 of 46 countries, accounting for 30.7% of infants unvaccinated with MCV during 2010, with either insufficient or no progress were classified as developing or least developed countries; 22 countries were located in Africa) (Table 2). Nonetheless, immunization coverage has improved substantially among developing or least developed countries since 2000.

**Table 2:** Listing of countries with either insufficient or no progress towards the GIVS coverage goal for the third dose of diphtheria and tetanus toxoid with pertussis vaccine (DTP3) and for the first dose of measles containing vaccine (MCV)

DTP3	MCV
Afghanistan*	Afghanistan*
Austria*	Austria*
Azerbaijan**	Azerbaijan**
Barbados*	Barbados*
Bolivia*	Benin*
Central African Republic**	Bolivia**
Chad*	Central African Republic**
Colombia*	Chad*
Comoros*	Colombia*
Costa Rica*	Comoros*
Democratic Republic of the Congo*	Costa Rica*
Dominican Republic*	Côte d'Ivoire**
Equatorial Guinea**	Cyprus*
Gabon**	Democratic Republic of the Congo*
Guinea**	Dominican Republic**
Guinea-Bissau**	Equatorial Guinea**
Haiti**	Gabon**
India*	Guinea**
Iraq**	Guinea-Bissau**
Latvia*	Haiti**
Lebanon**	Iraq**
Lesotho*	Lebanon**
Liberia*	Lesotho*
Madagascar**	Liberia*
Mali**	Madagascar*
Malta*	Mali**
Mauritania**	Malta**
Micronesia (Federated States of )*	Mauritania*
Mozambique**	Micronesia (Federated States of )*
Namibia*	Mozambique**
Palau*	Namibia*
Papua New Guinea**	Palau**
Philippines*	Papua New Guinea**
Rwanda*	Philippines*
Senegal**	Rwanda*
Solomon Islands*	Samoa*
Somalia*	Senegal**
South Africa**	Solomon Islands**
Swaziland*	Somalia*
Syrian Arab Republic*	South Africa*
Tuvalu*	Suriname*
Uganda**	Syrian Arab Republic*
Vanuatu**	Uganda**
Venezuela*	Vanuatu**
Zambia*	Venezuela*
	Yemen**

* Countries making insufficient progress towards GIVS coverage goal of 90%.

** Countries making no progress towards GIVS coverage goal of 90%.

Among 137 countries classified as developing or least developed during 2000, 54 countries attained DTP3 coverage ≥ 90% (average DTP3 coverage for developing countries, 75%; for least developed countries, 61%) and 50 countries attained MCV coverage ≥ 90% (average MCV coverage for developing countries, 73%; for least developed countries, 59%); by 2010, 80 of 138 developing or least developed countries reached 90% DTP3 coverage (average DTP3 coverage for developing countries, 85%; for least developed countries, 80%) and 76 countries reached 90% MCV coverage (average MCV coverage for developing countries, 86%; for least developed countries, 78%). Among 138 countries classified as developing or least developed in 2010, 40% (n = 56) of countries sustained DTP3 coverage ≥ 90% during the five year period 2006-2010 and 43% (n = 59) of countries sustained MCV coverage ≥ 90% during the period. (N.B. The World Bank country classification is reviewed and revised as needed on an annual basis; therefore, the number of countries classified as developing or least developed can change from year to year.)

### *GIVS Progress among Priority Countries*

Among 68 priority countries where more than 95% of all maternal and child deaths occur, DTP3 coverage ranged from 24% to 98% (median 68%) during 2000 and from 33% to 99% (median 84%) during 2010 (Additional File 1, Table S1). Sixteen priority countries had DTP3 coverage estimates < 70% during 2010. For MCV, coverage ranged from 24% to 99% during 2000 (median 71%) and from 46% to 99% during 2010 (median 83%) with 21 priority countries reporting MCV coverage < 70% during 2010 (Additional File 2, Table S2).

Among the 68 priority countries, accounting for more than three-quarters of surviving infants globally (77.9%; 100,408,000 surviving infants) and more than 90% of surviving infants unvaccinated with DTP3 (92.1%; 17,787,000) and MCV (91.5%; 17,474,000), 28 were identified as having made either insufficient or no progress towards reaching the GIVS goal of 90% coverage for DTP3; similarly, 28 of 68 countries made insufficient or no progress for MCV coverage. Among 34 priority countries identified with DTP3 coverage < 70% during 2000, DTP3 coverage remained below 70% for 14 countries in 2010, increased to 80-89% for 5 countries, and increased to ≥ 90% for 8 countries by 2010. Similarly for MCV, coverage remained below 70% during 2010 for 18 of 33 priority countries, increased to 80-89% for 5 countries, and increased to ≥ 90% for 4 countries with MCV coverage < 70% during 2000.

## Discussion

Levels and trends of immunization coverage are used to monitor the performance of immunization services at local, national and international levels; to guide eradication, elimination and control strategies for vaccine preventable diseases [8-10]; to identify areas of immunization systems that may require additional resources and focused attention [1,11]; and to inform decisions as to whether new vaccines should be introduced into national and local immunization systems [12]. Moreover, measles immunization coverage is one of the indicators for tracking progress towards achievement of the Millennium Development Goals [13].

Although coverage targets should be kept in perspective and not distract from technically appropriate and sustainable immunization programme activities [14], high levels of immunization coverage are important to prevent and control vaccine-preventable diseases. Our review of national routine immunization coverage estimates demonstrates that substantial progress has been made: more than three-quarters of countries, accounting for more than two-thirds of surviving infants globally in 2010, achieved at least 90% coverage during 2010 or were on track to achieve ≥ 90% coverage for DTP3 and MCV by 2015, and 118 countries maintained DTP3 coverage ≥ 90% for four of the first five years of GIVS. The data suggest that by 2015, 45 countries will not have reached the 90% target for DTP3 (46 countries for measles) if the trend identified for 2005-2010 is correct and maintained. These 45 countries account for an estimated two-thirds of surviving infants unvaccinated with DTP3, most are developing or least developed countries and roughly half are located in Africa. Thus, developing and least developed countries continue to struggle to attain and maintain high coverage levels. Moreover, we identified 28 of 68 priority countries with either insufficient or no progress towards reaching the GIVS goal of 90% coverage for DTP3 and for MCV coverage as of 2010; 22 of the 28 priority countries were located in Africa.

The role of GIVS to address the challenges of vaccination programmes has been described [1,2,15]. GIVS provides countries with a mechanism to identify critical areas and resource needs. While many of the activities supported by GIVS began prior to its development, there is some evidence that these strategies (e.g., RED [11], child health days [15], among others) are resulting in improved immunization system outcomes. Ultimately, GIVS will be measured by how well countries, particularly the developing and least developed countries, are able to reduce vaccine preventable deaths, the total costs of which have been estimated at US$76 (range: US$23-110) billion including US$49 billion for maintenance of current systems and $27 billion for scaling-up in order to attain the GIVS goal of reducing mortality due to vaccine preventable diseases by two-thirds by 2015 [16].

The review was based on the 2010 revision of the WHO and UNICEF estimates of national immunization coverage (completed in July 2011). The limitations of these data have been described [3]. Perhaps most importantly, the quality of the estimates is determined by the quality and availability of empirical data. Vaccination coverage is comparatively easy to measure and two methods - administrative reports and surveys - have been developed, each of which, when properly designed and implemented, provides accurate and reliable direct measures of coverage levels. Implemented jointly (using each measure for the same population), they provide a validation of coverage levels. However, both methods are subject to biases. In some instances, these may be identified and corrected. In no instance are the WHO and UNICEF estimates based on complete, consistent, multiple measures for an entire country and vaccine time series. In some instances the WHO and UNICEF estimates are based on complete administrative data validated by periodic or occasional consistent survey findings. In others, data are available from a single source - usually administrative data - and appear internally consistent over time and across vaccines, while in some countries, administrative data and survey results are inconsistent and in others the administrative time series is incomplete, internally inconsistent or both. The method also does not attempt to triangulate coverage levels with disease incidence data. For example, some countries may have high MCV coverage level estimates but suffer at the same time severe measles outbreaks.

The WHO and UNICEF estimates are limited by the absence of any articulation of uncertainty; as presented, they appear equally precise and certain. The uncertainty in the estimates is rooted in the accuracy and precision of the empirical data (described above) and in the choice and application of the heuristics. Because the estimates are not based on a probability sample and multiple measures are not considered as random variants of a single population measure, limiting the uncertainty to the amount of variation in the empirical data is a challenge.

It is also important to emphasize that the WHO and UNICEF estimates reflect (to every extent possible) coverage levels attained through routine immunization system. Some countries may implement immunization programme activities (e.g., child health days [15] or campaigns) outside of the routine immunization system that target children missed by routine immunization systems. As stated above, such doses are not included in the estimated coverage levels and actual doses administered would therefore be higher than estimated by the coverage estimates.

## Conclusions

Immunization, one of the most cost-effective public health interventions [17,18], has made impressive contributions to reducing child mortality. Though much progress has been achieved, if we are clear sighted in the global vision for the future, we will acknowledge that there is room for much improvement. The benefits of vaccination continue to elude many of the world's children (during 2010 an estimated 19.3 million infants did not receive the three doses of DTP [19]). Furthermore, large differences in immunization coverage observed here between countries are compounded by disparities within countries. At the mid-term of the Global Immunization Vision and Strategy, we observed more than three-quarters of countries either achieved ≥ 90% coverage by 2010 or were on track to achieve ≥ 90% coverage by 2015 for both DTP3 and MCV. Unfortunately, challenges remain among developing or least developed countries with one-third of these countries not attaining 90% coverage. Nearly a quarter of 68 priority countries made no progress during the first five years of GIVS. Although there have been enormous and increasingly successful efforts to address the global burden of vaccine-preventable diseases and to increase immunization coverage, opportunities remain to improve routine immunization coverage globally. While all countries must make efforts to sustain their programmes, special attention will need to be directed towards those countries that are unlikely to achieve 90% coverage by 2015.

## Competing interests

The authors declare that they have no competing interests.

## Authors' contributions

DWB - conceived the manuscript, participated in the review and preparation of WHO and UNICEF estimates of national immunization coverage, analysis, discussion of data, drafting the article, and read and approved the final manuscript; AB, MGD, RK - participated in the review and preparation of WHO and UNICEF estimates of national immunization coverage, analysis, discussion of data, correcting of the article, and read and approved the final manuscript; JV, JMOB - participated in discussion of data, correcting of the article, and read and approved the final manuscript.

## Pre-publication history

The pre-publication history for this paper can be accessed here:

http://www.biomedcentral.com/1471-2458/11/806/prepub

## Supplementary Material

Additional file 1 **Table S1**. Progress towards 90% coverage with three doses of diphtheria and tetanus toxoid with pertussis (DTP3) vaccine among 68 countries accounting for more than 95% of all maternal and child deaths Click here for file

Additional file 2 **Table S2**. Progress towards 90% coverage with measles containing vaccine (MCV) among 68 countries accounting for more than 95% of all maternal and child deaths Click here for file
