# Supplementary material for: A mid-term assessment of progress towards the immunization coverage goal of the Global Immunization Vision and Strategy (GIVS)
Source: BMC Public Health. 2011 Oct 14;11:806. doi: 10.1186/1471-2458-11-806 (PMC3210114; doi:10.1186/1471-2458-11-806)
Supplement: Additional file 2 — Table S2. Progress towards 90% coverage with measles containing vaccine (MCV) among 68 countries accounting for more than 95% of all maternal and child deaths [file 1471-2458-11-806-S2.DOC]

**Table S2.** Progress towards 90% coverage with measles containing vaccine (MCV) among 68 countries accounting for more than 95% of all maternal and child deaths

|  | MCV coverage (%) | | | Avg annual rate of change (%) | | Summary assessment of progress* |
| --- | --- | --- | --- | --- | --- | --- |
| Country or territory | 2000 | 2005 | 2010 | 2000-2005 | 2005-2010 |
| Afghanistan | 27 | 50 | 62 | 13.1 | 5.5 | Insufficient |
| Angola | 41 | 45 | 93 | 1.9 | 19.9 | On track |
| Azerbaijan | 67 | 67 | 67 | 0.0 | 0.0 | No progress |
| Bangladesh | 72 | 94 | 94 | 5.5 | 0.0 | On track |
| Benin | 70 | 61 | 69 | -2.7 | 3.1 | Insufficient |
| Bolivia | 84 | 89 | 79 | 1.2 | -2.9 | No progress |
| Botswana | 91 | 93 | 94 | 0.4 | 0.3 | On track |
| Brazil | 99 | 99 | 99 | 0.0 | 0.0 | On track |
| Burkina Faso | 59 | 84 | 94 | 7.3 | 2.9 | On track |
| Burundi | 76 | 87 | 92 | 2.7 | 1.4 | On track |
| Cambodia | 65 | 79 | 93 | 4.0 | 4.2 | On track |
| Cameroon | 49 | 68 | 79 | 6.8 | 3.8 | On track |
| Central African Rep. | 36 | 62 | 62 | 11.5 | 0.0 | No progress |
| Chad | 28 | 32 | 46 | 2.7 | 9.5 | Insufficient |
| China | 84 | 86 | 99 | 0.5 | 3.6 | On track |
| Congo | 34 | 56 | 76 | 10.5 | 7.9 | On track |
| Congo, Dem. Rep. | 46 | 61 | 68 | 5.8 | 2.8 | Insufficient |
| Côte d’Ivoire | 68 | 84 | 70 | 4.3 | -4.5 | No progress |
| Djibouti | 50 | 65 | 85 | 5.4 | 6.9 | On track |
| Egypt | 98 | 98 | 96 | 0.0 | -0.5 | On track |
| Equatorial Guinea | 51 | 51 | 51 | 0.0 | 0.0 | No progress |
| Eritrea | 76 | 95 | 99 | 4.6 | 1.0 | On track |
| Ethiopia | 52 | 59 | 81 | 2.6 | 8.2 | On track |
| Gabon | 55 | 55 | 55 | 0.0 | 0.0 | No progress |
| Gambia | 89 | 91 | 97 | 0.4 | 1.6 | On track |
| Ghana | 98 | 83 | 93 | -3.3 | 2.9 | On track |
| Guatemala | 86 | 93 | 93 | 1.6 | 0.0 | On track |
| Guinea | 42 | 51 | 51 | 4.0 | 0.0 | No progress |
| Guinea-Bissau | 71 | 76 | 61 | 1.4 | -5.3 | No progress |
| Haiti | 55 | 59 | 59 | 1.4 | 0.0 | No progress |
| India | 55 | 64 | 74 | 3.1 | 3.7 | On track |
| Indonesia | 74 | 77 | 89 | 0.8 | 3.7 | On track |
| Iraq | 87 | 69 | 73 | -4.5 | 1.4 | Insufficient |
| Kenya | 75 | 69 | 86 | -1.7 | 5.7 | On track |
| Korea, Dem. Rep. | 78 | 96 | 99 | 4.2 | 0.8 | On track |
| Lao People’s Dem. Rep. | 42 | 41 | 64 | -0.5 | 11.8 | On track |
| Lesotho | 74 | 85 | 85 | 2.8 | 0.0 | Insufficient |
| Liberia | 63 | 63 | 64 | 0.0 | 0.4 | Insufficient |
| Madagascar | 55 | 61 | 67 | 2.1 | 2.4 | Insufficient |
| Malawi | 73 | 82 | 93 | 2.4 | 3.2 | On track |
| Mali | 55 | 73 | 63 | 5.8 | -3.6 | No progress |
| Mauritania | 62 | 61 | 67 | -0.3 | 2.4 | Insufficient |
| Mexico | 96 | 96 | 95 | 0.0 | -0.3 | On track |
| Morocco | 93 | 97 | 98 | 0.8 | 0.3 | On track |
| Mozambique | 71 | 75 | 70 | 1.1 | -1.7 | No progress |
| Myanmar | 84 | 72 | 88 | -3.0 | 5.1 | On track |
| Nepal | 77 | 74 | 86 | -0.8 | 3.8 | On track |
| Niger | 37 | 47 | 71 | 4.9 | 10.9 | On track |
| Nigeria | 33 | 41 | 71 | 4.4 | 14.7 | On track |
| Pakistan | 59 | 78 | 86 | 5.7 | 2.5 | On track |
| Papua New Guinea | 62 | 63 | 55 | 0.3 | -3.3 | No progress |
| Peru | 97 | 80 | 94 | -3.8 | 4.1 | On track |
| Philippines | 80 | 92 | 88 | 2.8 | -1.1 | Insufficient |
| Rwanda | 74 | 89 | 82 | 3.8 | -2.0 | Insufficient |
| Senegal | 48 | 74 | 60 | 9.0 | -5.1 | No progress |
| Sierra Leone | 37 | 71 | 82 | 13.9 | 3.7 | On track |
| Somalia | 24 | 35 | 46 | 7.8 | 7.1 | Insufficient |
| South Africa | 72 | 64 | 65 | -2.3 | 0.4 | Insufficient |
| Sudan** | 58 | 69 | 90 | 3.5 | 6.9 | On track |
| Swaziland | 92 | 92 | 94 | 0.0 | 0.5 | On track |
| Tajikistan | 88 | 85 | 94 | -0.7 | 2.5 | On track |
| Togo | 58 | 70 | 84 | 3.8 | 4.7 | On track |
| Turkmenistan | 96 | 99 | 99 | 0.6 | 0.0 | On track |
| Uganda | 57 | 68 | 55 | 3.6 | -5.2 | No progress |
| Tanzania, U. Rep. | 78 | 91 | 92 | 3.1 | 0.3 | On track |
| Yemen | 71 | 76 | 73 | 1.4 | -1.0 | No progress |
| Zambia | 85 | 85 | 91 | 0.0 | 1.7 | On track |
| Zimbabwe | 75 | 66 | 84 | -2.5 | 6.2 | On track |

* “On track” indicates that the DTP3 coverage for 2010 is >/=90% or that coverage is < 90% with an average annual rate of increase for the period 2005-2010 necessary to reach 90% by 2015; “insufficient progress” indicates that the DTP3 coverage for 2010 is < 90% with an average annual rate of increase for the period 2005-2010 less than that necessary to reach 90% by 2015; “no progress” indicates that the DTP3 coverage for 2010 is < 90% with no change in coverage or an average annual decrease for the period 2005-2010 that falls short necessary to reach 90% by 2015.

** Due to the cession in July 2011 of the Republic of South Sudan from the Republic of the Sudan, and its subsequent admission to the UN on 14 July 2011, disaggregated data for Sudan and South Sudan as separate States are not yet available. Data presented are for Sudan pre-cession.

Note: The 68 priority countries listed here comprise a group focused on by the *Countdown to 2015: Tracking Progress in Maternal, Newborn and Child Survival* project, a global effort supported by academics, governments, international agencies, health care professional associations, donors and nongovernmental organizations.^6^

*Source:* WHO and UNICEF estimates of national immunization coverage, 2010 revision (July 2011)
